# Counteractive role of *Terminalia catappa* leaf extract on hematological and coagulation disturbance in Type 2 diabetic rats

**DOI:** 10.14202/vetworld.2023.1593-1599

**Published:** 2023-08-11

**Authors:** Franklyn Nonso Iheagwam, Precious Amaneshi Garuba, Olubanke Olujoke Ogunlana, Shalom Nwodo Chinedu

**Affiliations:** 1 Department of Biochemistry, Covenant University, PMB 1023, Ota, Ogun State, Nigeria; 2 Covenant University Public Health and Wellbeing Research Cluster, Covenant University, PMB 1023, Ota, Ogun State, Nigeria

**Keywords:** coagulation, diabetes, hematology, high-fat diet, streptozotocin-induced, *Terminalia catappa*

## Abstract

**Background and Aim:**

Diabetes mellitus is a leading cause of mortality worldwide associated with hyperglycemia-induced hematological aberrations and thromboembolic complications. This study aimed to explore the modulatory effect of *Terminalia catappa* leaf aqueous crude extract (TCLE) on hematological and coagulation disturbances in a Type 2 diabetic rat model.

**Materials and Methods:**

High-fat diet streptozotocin-induced diabetic rats were treated orally with 400 and 800 mg/kg body weight TCLE daily for 28 days. Full blood count, coagulation parameters, plasma calcium (Ca), and erythrocyte glycogen (GLYC) levels were assessed using standard procedures.

**Results:**

*Terminalia catappa* leaf aqueous crude extract treatment had a significant (p < 0.05) prolonging effect on clotting and bleeding times while increasing Ca, GLYC and mean corpuscular volume in diabetic rats. On the other hand, lymphocytes (LYM), platelet (PLT) count, mean PLT volume, neutrophil-LYM ratio (NLR), and PLT-LYM ratio (PLR) of TCLE-treated diabetic animals were significantly reduced (p < 0.05) compared with untreated diabetic animals. Lymphocyte, PLT count, NLR, and PLR correlated positively (p < 0.05) with plasma glucose, while a significant positive association was observed between Ca and GLYC. On the other hand, a strong negative association (p < 0.05) was observed between clotting time and fasting plasma glucose.

**Conclusion:**

These findings suggest that *T. catappa* leaf extract may be useful in reversing diabetic-mediated hematological anomalies due to its anticoagulant and anti-anemic activities.

## Introduction

Diabetes mellitus (DM) is a leading cause of mortality worldwide, with an abnormal rise in blood glucose levels as the hallmark [1, 2]. Type 2 DM (T2DM) has the highest incidence and prevalence in sub-Saharan Africa and requires urgent attention due to its impact on public health. It is associated with two critical factors, namely; impaired insulin production by pancreatic β-cells and reduced insulin sensitivity in peripheral tissues [1]. This brings about a pathophysiological change in systemic metabolic activities and regulation of major macromolecules, leading to severe hyperglycemia through a cascade of mechanisms [1, 3]. Uncontrolled T2DM is associated with cellular, metabolic, and blood disturbances, leading to micro and macrovascular complications [3].

Diabetics usually present altered immune systems and hematological characteristics on clinical evaluation, which correlates with various hematological abnormalities [4]. These hematological aberrations may manifest as immunological problems, coagulation dysfunction, and anemia contributing to cardiovascular diseases (CVD) [5]. Hematological dysfunction as a result of chronic hyperglycemia also leads to the formation of advanced glycation end products and increased production of reactive oxygen species, which then brings about platelet (PLT) hyperactivity, red blood cell (RBC), and endothelial dysfunction [6]. The high mortality observed in T2DM has been attributed to thrombosis, a concomitant effect of increased clotting factors and PLT activity [7]. Thrombohemorrhagic balance impairment in diabetics makes them susceptible to atherosclerosis, increased plaque rupture, and thromboembolic complications altering erythrocytes and PLTs architecture and fibrin networks degenerating quality of life [8].

A large portion of the Nigerian population uses medicinal plants rather than allopathic medications to treat and manage T2DM and its resultant vascular complications in the hope of getting better [9]. Studies have reported that ingested medicinal plants can alter hematological parameters [10]. Hence, hematological indicators may be crucial in evaluating the therapeutic effects of medicinal plants [11]. *Terminalia catappa* L. is a medicinal plant with antidiabetic properties [1]. *Terminalia catappa* leaf has been reported to improve the hematological profile and survival in *Betta* spp. [12]. The stem bark extract and its fractions were able to reverse hematologic alterations in three different chemical-induced diabetic models [13]. Insulin resistance (IR) induces vascular complications in T2DM by increasing the concentration of circulating inflammatory biomarkers, exacerbating RBC structural and morphological alteration while inducing PLT hyperactivity [14, 15]. The previous studies by Iheagwam *et al*. [1] and Iheagwam *et al*. [16] have shown that *T. catappa* leaf abrogates oxidative stress, inflammation, and IR in diabetic rats. Nonetheless, there is a paucity of information on the role of *T. catappa* leaf on diabetes-induced altered hematology and coagulation dysfunction.

This study aimed to explore the modulatory effect of *T. catappa* leaf aqueous crude extract (TCLE) on hematological and coagulation disturbances in a streptozotocin (STZ)-induced Type 2 diabetic rat model.

## Materials and Methods

### Ethical approval

All animal handling and experimental procedures were carried out following the animal research: Reporting of *in vivo* experiments (ARRIVE) guidelines for the care and use of laboratory animals approved by Covenant University Health and Research Ethics Committee (CHREC/031/2018).

### Study period and location

This study was undertaken from January 2018 to May 2018 at the animal house and laboratory of the Department of Biochemistry, Covenant University, Ota, Ogun State, Nigeria.

### Chemicals and reagents

Streptozotocin (≥98%) was obtained from Solarbio Science and Technology (Beijing, China) while other chemicals and solvents of analytical grade used in this study were purchased from Sigma-Aldrich Chemical Co. (St. Louis, USA).

### Plant collection and preparation of crude extract

Fresh mature *T. catappa* leaves were collected from trees on Covenant University campus, Nigeria. They were authenticated in the herbarium of the Forest Research Institute of Nigeria with code FHI 112775. The crude extract was prepared following the method of Iheagwam *et al*. [17]. Briefly, *T. catappa* leaves were washed, shade-dried, and marinated in purified water (5% w/v) after which the filtrate was concentrated to dryness in a rotary evaporator (Stuart RE 300/MS, Staffordshire, UK) to yield the TCLE.

### Animals and experimental care

Male Wistar rats (n = 30, 200 ± 20 g, age = 6–8 weeks) were used for this study. Experimental animals were acclimatized for 2 weeks before the experiment, provided nourishments, and maintained under standard light/dark cycle, room temperature, and humidity.

### Experimental design

Rats (n = 30) were randomly divided into five groups (n = 6):

- Group I, normal rats treated with distilled water (1 mL/kg body weight [BW]);
- Group II, diabetic rats treated with distilled water (1 mL/kg BW);
- Group III, diabetic rats treated with glibenclamide (10 mg/kg BW);
- Group IV, diabetic rats treated with TCLE (400 mg/kg BW); and
- Group V, diabetic rats treated with TCLE (800 mg/kg BW).

The experimental period and dosage determination were similar to the study of Iheagwam *et al*. [16]. Type 2 DM was induced using a low-dose STZ (30 mg/kg BW) and high-fat diet (HFD) following the protocol reported by Iheagwam *et al*. [1]. The treatment was administered by gastric intubation daily according to the experimental design for 4 weeks. After the experiment, animals were fasted overnight and anesthetized using xylazine/ketamine (1:10 v/v). Fresh blood was obtained through a cardiac puncture; a portion was placed in an ethylene diamine tetraacetate bottle for hematology parameters, while the rest was collected in a heparin tube and separated to obtain plasma for calcium (Ca) analysis.

### Coagulation analyses

#### Clotting time (CT)

Clotting time was evaluated following Ivy’s method [18]. The animal’s tail was cut to obtain a drop of blood on a glass slide. A stopwatch was started simultaneously before a pin was passed across the drop of blood at 15-s intervals until fibrin threads were noticed. Thereafter, the timer was stopped and recorded as the CT.

#### Bleeding time (BT)

Bleeding time was performed following the method reported by Ayodele *et al*. [8]. A proximal cut was made between 1 and 2 cm from the tail end to obtain blood spots on a blotting paper. A stopwatch was started simultaneously before blood spots were made at 15-s intervals until the bleeding stopped. Thereafter, the timer was stopped and recorded as the BT.

### Biochemical and hematological analyses

Plasma Ca was analyzed according to the Randox diagnostic kit (Crumlin, UK) instructions. Erythrocyte glycogen (GLYC) concentration was assessed following the protocol reported by Iheagwam *et al*. [16]. White blood cell count, lymphocyte (LYM), medium-ranged monocytes, eosinophils, basophils, blasts and other precursor white cells, granulocytes, hemoglobin (Hb), RBC count, hematocrit, mean corpuscular volume (MCV), mean corpuscular hemoglobin (MCH), MCH concentration, red cell distribution width, PLT count, plateletcrit, mean PLT volume (MPV), and PLT distribution width were evaluated in an automated hematology analyzer (Mindray Automated analyzer BC 3200, China). Neutrophil-LYM ratio (NLR) and PLT-LYM ratio (PLR) were calculated.

### Statistical analysis

Data obtained were analyzed using IBM statistical package for the social sciences statistics 23 (IBM Corp., New York, USA) with results presented as mean ± standard error of the mean (SEM). Values were considered statistically different at a 5% probability level using a one-way analysis of variance and Duncan’s multiple range test for *post hoc* analyses. Indices significantly altered by the diabetic state were assessed for association with parameters of hyperglycemia using Pearson’s correlation.

## Results

### *Terminalia catappa* leaf extract effect on coagulation and biochemical parameters

**Figure-1:**
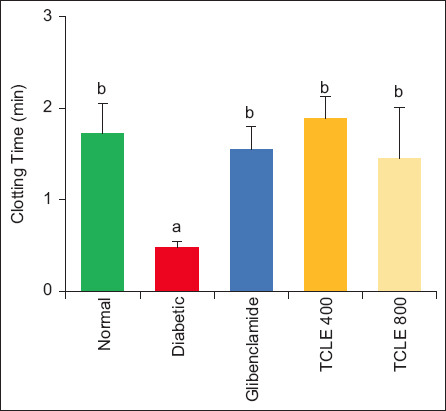
Effect of TCLE treatment on clotting time in high-fat diet/streptozotocin-induced diabetic rats. Bars are represented as mean ± standard error of the mean (n = 6). Bars with different superscripts are significantly different at p < 0.05. TCLE=*Terminalia catappa* leaf aqueous crude extract.

**Figure-2:**
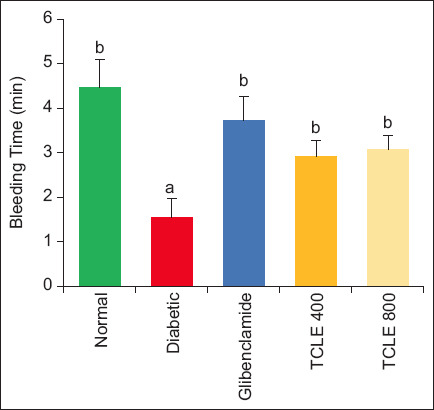
Effect of TCLE treatment on bleeding time in high-fat diet/streptozotocin-induced diabetic rats. Bars are represented as mean ± standard error of the mean (n = 6). Bars with different superscripts are significantly different at p < 0.05. TCLE=*Terminalia catappa* leaf aqueous crude extract.

The results of TCLE treatment on coagulation parameters are illustrated in Figures-1 and 2. A significant (p < 0.05) reduction in CT and BT was observed in HFD/STZ-induced diabetic rats compared with normal rats. These parameters were significantly (p < 0.05) restored to normal in diabetic rats treated with TCLE compared to untreated diabetic animals.

**Figure-3:**
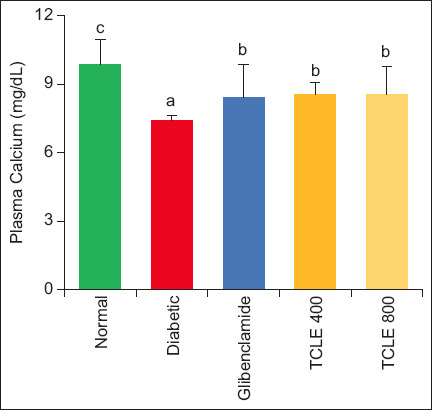
Effect of TCLE treatment on plasma calcium in high-fat diet/streptozotocin-induced diabetic rats. Bars are represented as mean ± standard error of the mean (n = 6). Bars with different superscripts are significantly different at p < 0.05. TCLE=*Terminalia catappa* leaf aqueous crude extract.

**Figure-4:**
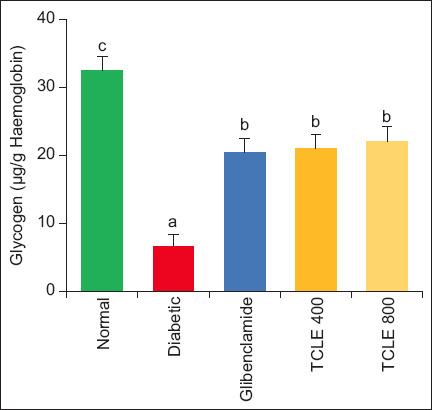
Effect of TCLE treatment on erythrocyte glycogen in high-fat diet/streptozotocin-induced diabetic rats. Bars are represented as mean ± standard error of the mean (n = 6). Bars with different superscripts are significantly different at p < 0.05. TCLE=*Terminalia catappa* leaf aqueous crude extract.

Compared to normal rats, Ca concentration and GLYC stores were depleted (p < 0.05) in diabetic rats. Treatment with TCLE and glibenclamide significantly increased Ca and GLYC concentration in diabetic rats compared with untreated diabetic rats. The values of Ca and GLYC in the treatment groups were significantly (p < 0.05) lower than those in the normal group (Figures-3 and 4).

### *Terminalia catappa* leaf extract effect on hematological parameters

In Table-1, diabetes onset led to a significant (p < 0.05) increase in LYM. Administration of TCLE significantly reduced diabetic rats’ LYM count compared with the normal and glibenclamide-treated rats. All other leukocyte indices in the experimental groups were not altered (p > 0.05) after diabetes induction compared to the sham group.

**Table-1:** Effect of TCLE treatment on leukocyte indices in HFD/STZ-induced diabetic rats.

Parameter	Normal	Diabetic	Glibenclamide	TCLE 400 mg/kg	TCLE 800 mg/kg
WBC (×10^12^/L)	5.48 ± 0.48^a^	6.86 ± 0.60^a^	4.89 ± 0.96^a^	6.03 ± 1.34^a^	4.54 ± 1.00^a^
LYM (×10^9^/L)	1.46 ± 0.33^a^	4.30 ± 0.96^b^	2.52 ± 0.87^a^	1.81 ± 0.66^a^	1.70 ± 0.68^a^
MID (×10^9^/L)	2.28 ± 0.45^a^	2.40 ± 0.40^a^	1.80 ± 0.57^a^	2.76 ± 0.98^a^	2.58 ± 0.46^a^
GRN (×10^9^/L)	1.74 ± 0.26^a^	0.92 ± 0.20^a^	2.62 ± 1.16^a^	1.46 ± 0.56^a^	2.64 ± 1.05^a^

Data are represented as mean ± SEM (n=6). Values with different superscripts across a row significantly differ at P < 0.05. TCLE=T*erminalia catappa* leaf aqueous crude extract, HFD=High-fat diet, STZ=Streptozotocin, WBC=White blood cells, LYM=Lymphocytes, MID: Medium-ranged monocytes, eosinophils, basophils, blasts, and other precursor white cells, GRN=Granulocyte

Among all evaluated erythrocyte indices, only MCV was significantly (p < 0.05) reduced in the diabetic group compared with the normal group. Diabetic rats treated with TCLE exhibited a marked increase (p < 0.05) in MCV when compared to untreated diabetic rats, with values similar to those obtained for normal and glibenclamide groups (Table-2).

**Table-2:** Effect of TCLE treatment on erythrocyte indices in HFD/STZ-induced diabetic rats.

Parameter	Normal	Diabetic	Glibenclamide	TCLE 400 mg/kg	TCLE 800 mg/kg
Hb (g/dL)	14.62 ± 0.43^a^	13.98 ± 1.12^a^	14.24 ± 0.41^a^	16.14 ± 0.49^a^	14.70 ± 1.22^a^
RBC (×10^7^/L)	7.54 ± 0.31^a^	6.51 ± 0.16^a^	6.68 ± 0.89^a^	7.51 ± 0.22^a^	7.68 ± 0.92^a^
HCT (%)	49.26 ± 3.46^a^	49.84 ± 1.77^a^	47.38 ± 6.10^a^	69.88 ± 16.04^a^	50.74 ± 7.24^a^
MCV (fL)	76.66 ± 3.76^a^	66.46 ± 2.59^b^	71.14 ± 3.02^a^	73.44 ± 4.09^a^	75.12 ± 2.58^a^
MCH (pg/cell)	23.14 ± 2.25^a^	19.00 ± 1.05^a^	21.58 ± 1.44^a^	20.56 ± 0.77^a^	19.64 ± 1.28^a^
MCHC (g/dL)	30.10 ± 1.92^a^	28.60 ± 0.72^a^	30.48 ± 2.00^a^	28.50 ± 1.15^a^	30.74 ± 3.25^a^
RCDW - coefficient variation (%)	20.24 ± 1.17^a^	18.78 ± 0.93^a^	18.14 ± 0.49^a^	16.52 ± 2.12^a^	19.48 ± 0.84^a^
RCDW - standard deviation (fL)	41.36 ± 2.58^a^	39.04 ± 3.05^a^	37.14 ± 1.82^a^	37.74 ± 1.58^a^	40.94 ± 3.19^a^

Data are represented as mean ± SEM (n=6). Values with different superscripts across a row are significantly different at p *<* 0.05. TCLE=T*erminalia catappa* leaf aqueous crude extract, HFD=High-fat diet, STZ=Streptozotocin, Hb=Hemoglobin, RBC=Red blood cells, HCT=Hematocrit, MCV=Mean corpuscular volume, MCH=Mean corpuscular hemoglobin, MCHC=Mean corpuscular hemoglobin concentration, RCDW=Red cell distribution width

Platelet and MPV were significantly raised (p < 0.05) in the diabetic groups, while other thrombocyte indices were not changed by the diabetic state and treatments compared with the normal group (Table-3). Increased PLT and MPV in the diabetic rats were significantly (p < 0.05) reduced in the TCLE- and glibenclamide-treated groups to normal when compared with the diabetic group (Table-3).

**Table-3:** Effect of TCLE treatment on thrombocyte indices in HFD/STZ-induced diabetic rats.

Parameter	Normal	Diabetic	Glibenclamide	TCLE 400 mg/kg	TCLE 800 mg/kg
PLT count (×10^12^/L)	551.40 ± 55.47^a^	720.60 ± 93.02^b^	578.60 ± 99.10^a^	583.40 ± 17.27^a^	576.20 ± 94.55^a^
MPV (fL)	9.98 ± 0.45^a^	19.20 ± 0.28^b^	10.58 ± 0.53^a^	6.10 ± 1.24^a^	8.92 ± 0.61^a^
PDW (%)	15.74 ± 0.11^a^	15.60 ± 0.19^a^	16.14 ± 0.23^a^	15.94 ± 0.18^a^	16.24 ± 0.25^a^
PCT (%)	0.55 ± 0.06^a^	0.50 ± 0.02^a^	0.53 ± 0.05^a^	0.56 ± 0.03^a^	0.60 ± 0.05^a^

Data are represented as mean ± SEM (n=6). Values with different superscripts across a row are significantly different at p *<* 0.05. TCLE=*Terminalia catappa* leaf aqueous crude extract, HFD=High-fat diet, STZ=Streptozotocin, PLT=Platelet, MPV=Mean platelet volume, PDW=Platelet distribution width, PCT=Plateletcrit

**Figure-5:**
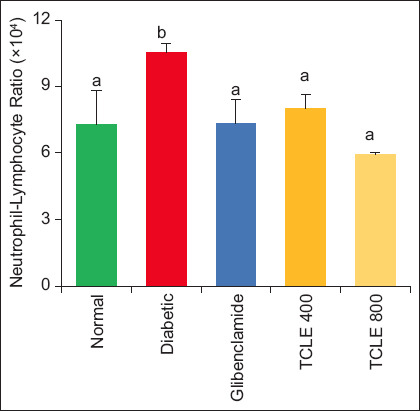
Effect of TCLE treatment on the neutrophil-lymphocyte ratio in high-fat diet/streptozotocin-induced diabetic rats. Bars are represented as mean ± standard error of the mean (n = 6). Bars with different superscripts are significantly different at p < 0.05. TCLE=*Terminalia catappa* leaf aqueous crude extract.

**Figure-6:**
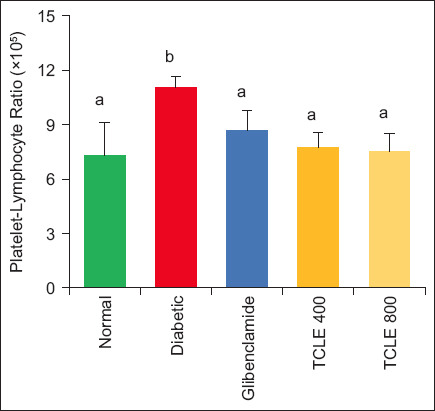
Effect of TCLE treatment on the platelet-lymphocyte ratio in high-fat diet/streptozotocin-induced diabetic rats. Bars are represented as mean ± standard error of the mean (n = 6). Bars with different superscripts are significantly different at p < 0.05. TCLE=*Terminalia catappa* leaf aqueous crude extract.

Administration of 400 and 800 mg/kg BW TCLE in diabetic rats significantly (p < 0.05) reduced NLR and PLR compared to the diabetic group. These indices initially increased significantly (p < 0.05) after induction of diabetes when compared with normal rats (Figures-5 and 6).

### Association between assessed indices and parameters of hyperglycemia

Lymphocyte (0.78), PLT (0.83), NLR (0.81), and PLR (0.79) exhibited a significant (p < 0.05) strong positive correlation with fasting blood glucose (FBG), while a strong negative correlation was observed for CT (−0.75, p < 0.05). In relation to erythrocyte GLYC, only Ca (0.86) was associated significantly (p < 0.05) in a positive manner (Table-4).

**Table-4:** Association between significantly altered parameters and hyperglycemia parameters in HFD/STZ-induced diabetic rats.

Parameters	Fasting blood glucose		Glycogen	
	
	R^2^	p-value	R^2^	p-value
Ca	−0.73	0.08	**0.86**	0.00
CT	**−0.75**	0.04	0.65	0.15
BT	−0.67	0.13	0.22	0.60
NLR	**0.81**	0.03	−0.56	0.22
PLR	**0.79**	0.04	−0.75	0.07
LYM	**0.78**	0.04	−0.76	0.06
MCV	−0.40	0.39	0.62	0.17
PLT	**0.83**	0.01	−0.75	0.07
MPV	0.67	0.13	−0.52	0.27

Correlation values in bold are significantly different at p *<* 0.05 (2-tailed). TCLE=*Terminalia catappa* leaf aqueous crude extract, HFD=High-fat diet, STZ=Streptozotocin, Ca=Calcium, CT=Clotting time, BT=Blotting time, NLR=Neutrophil-lymphocyte ratio, PLR=Platelet-lymphocyte ratio, LYM=Lymphocyte, MCV=Mean corpuscular volume, PLT=Platelet count, MPV=Mean platelet volume

## Discussion

Diabetic conditions, especially T2DM and obesity, are associated with a procoagulant state that induces thromboembolic complications, increasing the mortality rate of diabetics and patients with CVD [19, 20]. Microcapillary embolization, thrombus formation, and CVD development are triggered during the diabetic state [21]. Consequently, using anticoagulants in addition to oral hypoglycemic drugs and other diabetic therapies is important in reducing thrombosis risk in diabetic patients. Both CT and BT are important indices used to evaluate the progression of diabetic-induced thromboembolism [8]. The former measures an anomaly in the intrinsic pathway, while the latter measures the vascular and PLT hemostasis responses [22]. The lowered CT and BT in the diabetic rats may result from the increase in PLT and MPV, the decrease in hyperglycemia, and the increase in GLYC stores. This agrees with the previous reports by Nnah [7], Ayodele *et al*. [8], and Fidele *et al*. [23], who reported the negative association observed between CT and FBG. Hyperglycemia dysregulates signaling pathways responsible for PLT activation and aggregate response, increasing PLT [7]. Higher MPV suggests larger PLT size as such, there is more secretion of thromboxane A2, serotonin, and β-thromboglobulin, leading to better PLT aggregation [21]. *Terminalia catappa* leaf aqueous crude extract’s ability to increase CT and BT while reducing PLT and MPV might suggest a possible anticoagulant and fibrinolytic activity. *Terminalia catappa* leaf aqueous crude extract contains active principles such as benzofuranone, flavonoids, and octadecanoic acid that decrease the production of thromboxane A2, slowing down PLT aggregation and thereby increasing BT and CT [24, 25]. This finding is corroborated by similar studies that show medicinal plants improve hyperglycemia-altered coagulation signaling and thrombocyte indices in addition to BT/CT and PLT inverse relationship [23, 25, 26]. Nonetheless, a contrary finding reported no association between PLT and FBG [8, 27].

Calcium is required in blood coagulation for tenase and prothrombinase complex formation to activate prothrombin and transform fibrinogen into fibrin. The observed decrease in diabetic animals suggests that hypercoagulant signaling utilizes a large amount of systemic Ca, depleting Ca reserve in the process [8]. An increase in Ca concentration in experimental animals by TCLE suggests reversing the procoagulant state. Erythrocytes and hepatocytes share similar properties in relation to glucose transport and storage due to their role in glucose homeostasis [28]. The decrease in GLYC during diabetic onset could be attributed to IR, making glucose unavailable for utilization by erythrocytes [16]. There are reports of little to no glycogen accumulation in the erythrocytes of diabetics, substantiating the findings of this study by Malaisse [29] and Segers *et al*. [30]. Erythrocyte GLYC increase on TCLE treatment would suggest the reversal of IR, hence, glucose utilization and storage.

Streptozotocin possesses a cytotoxic effect in the bone marrow, leading to a suppressed immune system [31]. This cytotoxic effect might be associated with the observed LYM increase in diabetic rats. The reduction of LYM in TCLE experimental groups could be attributed to the immune-enhancing activity of the extract due to its phytoconstituents. In the reports of Oyedemi *et al*. [32] and Rashid *et al*. [31], LYM levels were increased upon administration of extracts, contrary to our findings. The association between LYM and FBG in this study is in discordance with the previous finding of Arkew *et al*. [5], who reported no association. However, Krisnamurti *et al*. [27] reported a correlation between these variables. The reduction of MCV in the diabetic animals indicates a possible risk of anemia. This predicament, particularly the hypochromic type, is associated with T2DM due to a drop in iron content, erythrocyte oxidative injury, and formation of glycated end products with erythrocytes [1, 33]. The restorative ability of TCLE to normal levels would suggest the anti-anemic property of the extract attributed to the antioxidant property similar to other reports by Iheagwam *et al*. [1], Chinedu-Ndukwe *et al*. [33] and Çelik *et al*. [34].

The association between T2DM and chronic inflammation induces diabetic microangiopathy [35]. During T2DM onset, low-degree chronic inflammation leads to hypersecretion of pro-inflammatory biomarkers elevating neutrophil count. The rise in NLR in diabetic rats underlies a possible elevation of inflammation and circulating inflammatory cytokines [36]. Like NLR, PLR is a potential inflammatory marker and predictor of diabetic microvascular complications [37]. Studies have also reported the rise in NLR and PLR during diabetes, corroborating this study’s findings [38–40]. Neutrophil-LYM ratio decrease in TCLE-administered rats may be due to the ability of the extract to truncate neutrophil activation and decrease the release of neutrophil proteases, which is upregulated during T2DM [41]. Furthermore, a decrease in both NLR and PLR in the treatment groups could result from TCLE reduction of LYM. The strong association between NLR and PLR with FBG gives credence to the fact that both ratios are predictors and prognostic risk markers of T2DM and its complications [42]. Contrary to our findings, a negative correlation was reported between PLT and FBG, while a positive correlation was observed for MPV and FBG [21].

## Conclusion

The findings signify that *T. catappa* leaf extract leaf may be useful in reversing diabetic-mediated hematological anomalies. Thus, TCLE may possess anticoagulant and anti-anemic activities useful for managing thrombotic disorder in the diabetic state. Prospective studies can be conducted to ascertain the anticoagulant and anti-anemic mechanism of action and the phytoprinciple(s) responsible.

## Authors’ Contributions

FNI, OOO, and SNC: Conceptualized the study. FNI: Designed the methodology, analyzed, and interpreted the result. FNI and PAG: Carried out the experiments and wrote the first draft of the manuscript. All authors have read, reviewed, and approved the final manuscript.
